# *Equid herpesvirus-1* Distribution in Equine Lymphoid and Neural Tissues 70 Days Post Infection

**DOI:** 10.3390/pathogens10060707

**Published:** 2021-06-05

**Authors:** Susanna Samoilowa, Kim S. Giessler, Carlos E. Medina Torres, Gisela Soboll Hussey, Allison Allum, Robert Fux, Christin Jerke, Matti Kiupel, Kaspar Matiasek, Dodd G. Sledge, Lutz S. Goehring

**Affiliations:** 1 Equine Hospital, Division of Medicine and Reproduction, Center for Clinical Veterinary Medicine, Ludwig-Maximilians University, 80539 Munich, Germany; giessle1@msu.edu (K.S.G.); c.medina@pferdeklinik-leichlingen.de (C.E.M.T.); Christin.jerke@stuaau.bwl.de (C.J.); Goehring@pferd.vetmed.uni-muenchen.de (L.S.G.); 2 Department of Pathobiology and Diagnostic Investigation, College of Veterinary Medicine, Michigan State University, East Lansing, MI 48824, USA; husseygi@msu.edu (G.S.H.); allison10allum@gmail.com (A.A.); kiupel@msu.edu (M.K.); 3 Division of Virology, Institute for Infectious Diseases and Zoonoses, Department of Veterinary Sciences, Ludwig-Maximilians University, 80539 Munich, Germany; robert.fux@lmu.de; 4 Veterinary Diagnostic Laboratory, College of Veterinary Medicine, Michigan State University, Lansing, MI 48824, USA; sledged@msu.edu; 5 Section of Clinical and Comparative Neuropathology, Centre for Clinical Veterinary Medicine, Ludwig-Maximilians Universitaet München, 80539 Munich, Germany; Kaspar.matiasek@neuropathologie.de

**Keywords:** EHV-1, herpesviruses, horse, latency, trigeminal ganglia, lymphoid tissue

## Abstract

Equid herpesvirus-1 (EHV-1) causes respiratory disease, abortion and myeloencephalopathy in horses worldwide. As member of the *Alphaherpesvirinae*, latency is key to EHV-1 epidemiology. EHV-1 latent infection has been detected in the trigeminal ganglion (TG), respiratory associated lymphoid tissue (RALT) and peripheral blood mononuclear cells (PBMC) but additional locations are likely. The aim of this study was to investigate the distribution of viral DNA throughout the equine body. Twenty-five horses divided into three groups were experimentally infected via intranasal instillation with one of three EHV-1 viruses and euthanized on Day 70, post infection. During necropsy, TG, various sympathetic/parasympathetic ganglia of head, neck, thorax and abdomen, spinal cord dorsal root ganglia, RALT, mesenteric lymph nodes, spleen and PBMC of each horse were collected. Genomic viral loads and L-(late) gene transcriptional activity in each tissue and PBMC were measured using qPCR. In addition, immunohistochemistry (IHC) was applied on neural parenchyma tissue sections. EHV-1 DNA was detected in many neural and lymphoid tissue sections, but not in PBMC. L-gene transcriptional activity was not detected in any sample, and translational activity was not apparent on IHC. Tissue tropism differed between the Ab4 wild type and the two mutant viruses.

## 1. Introduction

Equid herpesvirus-1 (EHV-1), a member of the Alphaherpesvirinae subfamily, causes respiratory disease, abortion, equid herpesvirus-associated myeloencephalopathy (EHM), chorioretinopathy, and infection of gonads of the intact male following a cell-associated viremia in peripheral blood mononuclear cells (PBMC) [1]. EHV-1 is typically spread by direct nose-to-nose contact followed by viral replication in the upper respiratory tract epithelium. This active lytic infection includes viremia and spread to the vascular endothelia of secondary sites of infection. After or even during the lytic phase, EHV-1 prepares for its characteristic chronic-persistent phase. This likely life-long infection phase is known as latent infection. In contrast to lytic infection, where transcriptional activity of the entire viral genome is fully upregulated, transcriptional activity during latency is likely limited to a single region known as the immediate early gene (IE) [2]. Current thoughts are that a latent infection is established early in life during first-time respiratory tract infection with EHV-1. It has been shown that the trigeminal ganglion (TG) is a site of EHV-1 latency that is likely reached via retrograde transportation along nerve axons [3,4]. In addition to the TG, EHV-1 latency has also been shown in the mandibular and retropharyngeal lymph node as part of the respiratory tract-associated lymphoid tissue (RALT), as well as in randomly collected circulating CD8+ T-lymphocytes [4,5]. However, we have recently provided evidence that cell-associated viremia might also be the vehicle of additional latency establishment at other sites by Day 30 post-infection (p.i.) [6]. In other members of the Alphaherpesvirinae, such as the Feline herpesvirus-1 (FeHV-1) and the human Varicella-Zoster Virus (VZV) additional latency sites have been detected at several sites of neural parenchyma. For the Bovine herpesvirus-1 (BoHV-1) and the Suid herpesvirus-1 (SHV-1) additional sites have been detected in lymphoid tissue [7,8,9,10,11,12,13]. We postulate that cell-associated viremia in PBMC is likely the carrier vehicle to other lymphatic or neural tissue sites in addition to TG and RALT.

In 2006, Nugent et al. showed a single nucleotide point (SNP) mutation in the EHV-1 DNA polymerase gene at position 2254 resulting in an amino acid switch (N vs. D752). The D-variants, retrospectively, have shown to be more often associated with clinical myelopathy than N-variants. This might be explained by a higher magnitude and duration of viremia of the D-variants [14]. Furthermore, EHV-4 is a closely related (equine alpha-) herpesvirus to EHV-1. Both viruses have a type-specific glycoprotein D (gD) in their respective envelopes, which is important for effective viral cell-to-cell spread. The capacity of EHV-1 gD for cell-to-cell transmission is thought to be greater than that for EHV-4 [15]. Therefore, we established three experimental infection groups for the present study. Group one horses were infected with the EHV-1 Ab4 wild-type (WT) strain with confirmed D752. Group two horses were infected with an EHV-1 Ab4 viral strain mutant where D was switched with N in the polymerase gene (EHV-1 Ab4 N752). Group three horses were infected with an Ab4 strain where the EHV-1 gD gene sequence was exchanged with the corresponding EHV-4 gD sequence (EHV-1 Ab4 gD4). Clinical and immunological data following infection with the different viruses has already been described previously [16].

Horses from all three groups were subsequently euthanized at 70 days post infection (dpi). The objective of the current study was to investigate the distribution of viral genomic DNA into various lymphoid and neural tissues by Day 70 p.i. and to investigate whether there is a difference in viral genomic DNA distribution pattern of EHV-1 Ab4 WT, the polymerase mutant (EHV-1 Ab4 N752) and the gD EHV-1/EHV-4 replacement mutant (EHV-1 Ab4 gD4). Our hypothesis was that there will be a wider distribution of EHV-1 beyond the currently accepted paradigm and that differences will exist between viral mutants.

## 2. Results

### 2.1. Clinical Signs, Nasal Viral Shedding and Viremia

The clinical signs, nasal viral shedding and viremia in the three groups were described elsewhere [16]. Briefly, Ab4 N752 infected horses showed the most severe signs of respiratory disease followed by Ab4 WT infected horses. Ab4 gD4 infected horses showed only mild respiratory disease. In contrast EHM (three out of eight horses) and a classical bi-phasic fever response was only observed in the Ab4 WT infected group. Furthermore, Ab4 WT infected horses presented significant higher viremia and shedding levels, while Ab4 gD4 infected horses shed significantly less virus and showed a late onset of viremia.

Two of the eight horses of the Ab4 WT infected group developed severe neurologic signs compatible with EHM, and were euthanized by Day 10 p.i. Therefore, tissue samples from only 23 horses were available in this study. In addition, 26 out of 299 tissue sections were lost during intercontinental transit between participating institutions.

### 2.2. Detection of EHV-1 Genomic DNA

**Figure 1:**
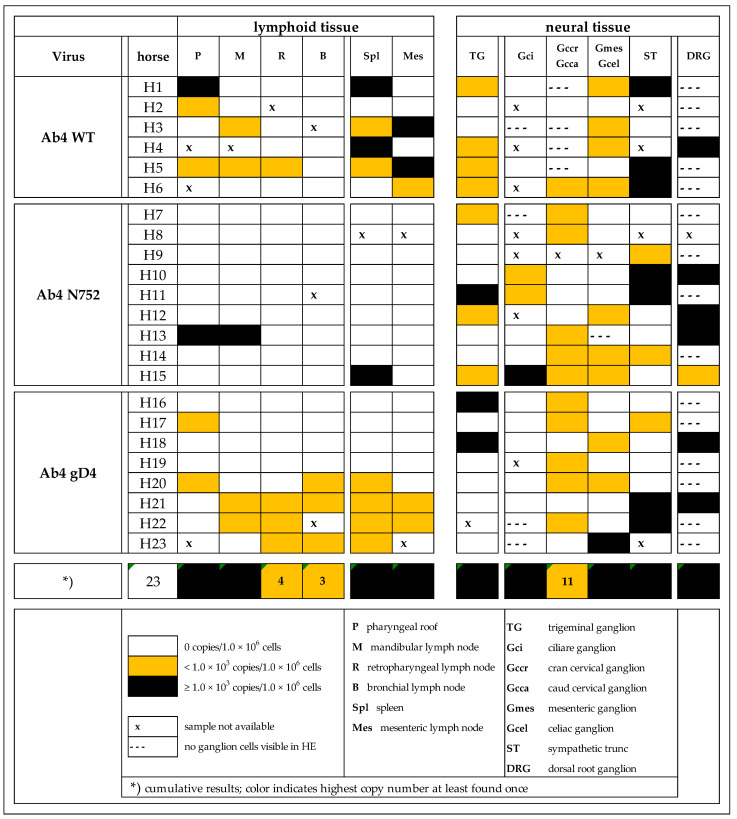
Distribution of EHV-1 genomic DNA in lymphoid and neural tissues [EHV-1 gB copies/1 × 10^6^ host cells] of yearling horses collected 70 days post infection with either EHV-1 Ab4 WT, Ab4 N752 or Ab4 gD4.

Many of the neural and lymphoid samples were viral genome (gDNA) positive, including tissue locations, that have not previously been described as viral sites of latency or persistence. A summary of viral loads in different tissue types from all groups is presented in Figure 1. Results are reported in three categories; no signal (undetectable during three separate efforts), <10^3^ viral copies/1 × 10^6^ host cells (low), or >10^3^ viral copies/1 × 10^6^ (high).

All PBMC samples but one collected on the day of euthanasia were negative for viral gDNA. The positive sample measured ‘low positive’ originated from the Ab4 WT infected group (data not shown). Furthermore, the most obvious finding when comparing groups of infected horses was a significant difference in tissue tropism in the Ab4 N752 infected group with a paucity of gDNA in lymphoid tissue when compared to the other two groups.

Sample quality with regard of target cells (lymphocytes or neuronal cell bodies) was good (tissue of interest/target cells present) in lymphoid tissues. However, in neural tissue samples, especially in dorsal root ganglia (DRG) and some of the sympathetic and parasympathetic (S/PS) tissues, sample quality was ‘moderate’ (tissue of interest/target cells partially present) or ‘poor’ (tissue of interest/target cells absent). In contrast, finding neuronal cell bodies in TG and sympathetic trunk (ST) was easy, as these are very distinct and macroscopically easy to recognize anatomical structures. TG consistently contained islands of nerve cell bodies and cut sections of cell nuclei (an estimated 1000–5000 cell bodies per cut section). Grossly suspected DRG samples contained nerve cell bodies in only seven of 22 samples (approximately 100–200 cells counted manually). Of the available 79 S/PS tissues, 70 contained nerve cell bodies (approximately 100–200 cells manually counted). Due to the cranial cervical ganglion’s close proximity to the respiratory epithelium-lined guttural pouch (an enlargement of the tuba auditiva and a structure exclusive to the horse), samples occasionally contained subepithelial (primary) lymph follicles in addition to the ganglionic nerve cell bodies. Using our scoring key (Table S1) to determine cyto-histopathological changes, most neural tissue samples (79 of 99) showed a mild or moderate lymphocytic/histiocytic cellular infiltrate. Of these, 71 were categorized as ‘mild’, and eight as ‘moderate’ infiltration. All ‘moderate’ infiltrates were detected in the mesenteric/coeliac ganglia or ST. As we suspected a potential contaminating role of viral gDNA positive lymphocytes/monocytes, we explored, if the degree of infiltration corresponds with positive viral gDNA outcome. As half of the samples were gDNA positive with four fifths showing a category ‘mild’ infiltrates, there was no evidence for a statistically significant positive correlation (*p >* 0.05).

**Figure 2:**
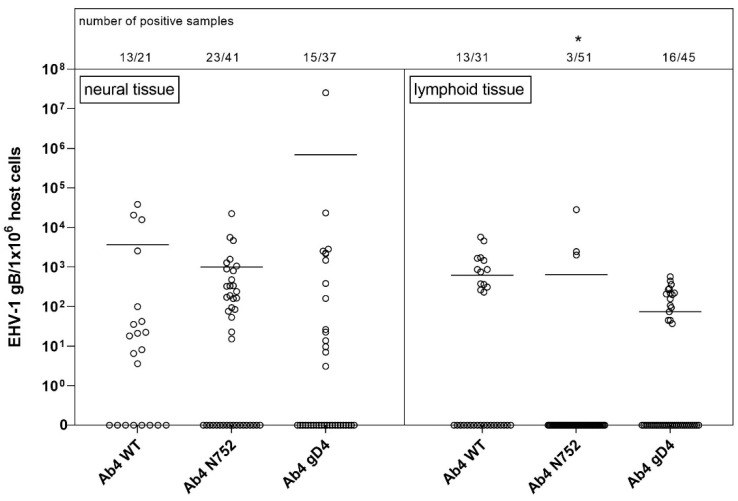
Viral loads (EHV-1 gB genome equivalents/1 × 10^6^ host cells) in neural (left panel) vs. lymphoid (right panel) tissue of EHV-1 infected yearling horses 70 dpi. Horses were infected with either EHV-1 Ab4 WT, Ab4 N752 or Ab4 gD4. Each data point (open circle) represents a tissue sample of an individual horse. An asterisk indicates significant lower affinity compared among and within groups (*p* < 0.005). The affinity is defined by the number of positive samples in combination with viral loads. Bars represent the mean. Note: data points at the 0-line (x-axis) represent samples where EHV-1 DNA was not detected despite neuronal cells or lymphocytes having been observed microscopically.

Figure 2 shows the viral (gB) copies per million (host) cells in all neural vs. lymphoid tissues combined for the three groups. Note that one cut section collected from lymphoid tissue probably contained several log-folds greater number of target cells (lymphocytes/monocytes) that are likely to carry viral DNA when compared to a neural tissue section that contained a range of other (support) cells besides neuronal cell bodies. Figure 2 also shows the low detection frequency of viral DNA in any lymphoid tissue of Ab4 N752 infected group. Only three lymphoid tissue samples out of 51 were positive for EHV-1 gDNA. Interestingly though, one of these three samples had very high copy numbers, showing an almost similar mean of viral loads when compared to the other groups. However, this group still differed significantly from the other groups. This difference results from the comparison of the affinity, that takes into consideration not only the viral load but also the number of positive samples. 

#### 2.2.1. Lymphoid Tissue

**Figure 3:**
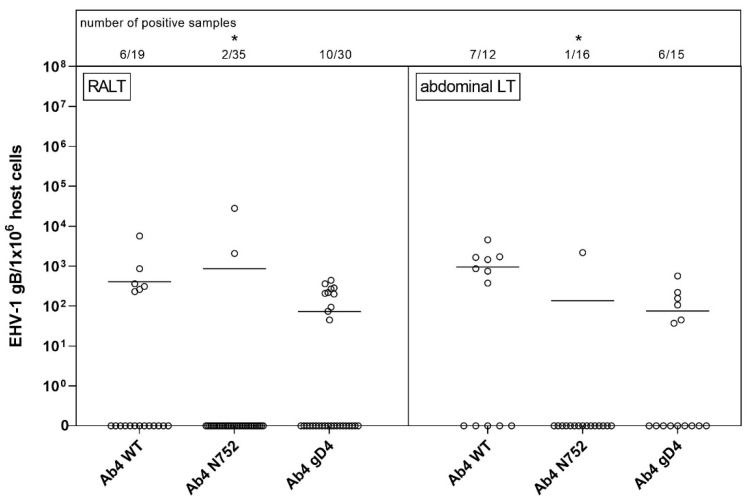
Viral loads (EHV-1 gB genome equivalents/1 × 10^6^ host cells) in respiratory-tract associated lymphoid tissue (RALT, left panel) vs. abdominal lymphoid tissue (abdominal LT, right panel) of EHV-1 infected yearling horses 70 dpi. Horses were infected with either EHV-1 Ab4 WT, Ab4 N752 or Ab4 gD4. Each data point (open circle) represents a tissue sample of an individual horse. An asterisk indicates significant lower affinity compared among groups (*p* < 0.005). The affinity is defined by the number of positive samples in combination with viral loads. Bars represent the mean. Note: data points at zero-line (x-axis) represent samples where EHV-1 DNA was not detected despite neuronal cells or lymphocytes having been observed microscopically.

Overall, the amount of positive samples for RALT was 18 of 23 and for abdominal lymphoid tissues 13 of 23 (Figure 3). Of 23 horses a total, viral gDNA was detected in the pharyngeal roof of six horses, as well as in the mandibular, retropharyngeal and bronchial lymph nodes of five, four, and three horses, respectively. Nine and five horses were positive in the spleen and mesenteric lymph node, respectively. A ‘high’ viral gDNA count was found three times in the spleen, two times in the pharyngeal roof and the mesenteric lymph node, and in one of the mandibular lymph nodes. Although the mean copy numbers were comparable between groups, there were significantly fewer viral gDNA positive samples (3/51) in the Ab4 N752 group even though this group of horses had a total of nine horses.

#### 2.2.2. Neural Tissue

**Figure 4:**
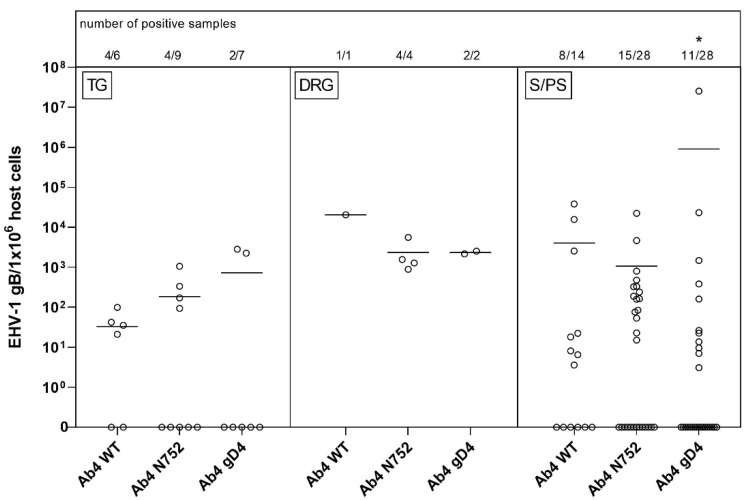
Viral loads (EHV-1 gB genome equivalents/1 × 10^6^ host cells) in trigeminal ganglia (TG, left panel) vs. dorsal root ganglia (DRG, middle panel) vs. sympathetic/parasympathetic ganglia (S/PS, right panel) of EHV-1 infected yearling horses 70 dpi. Horses were infected with either EHV-1 Ab4 WT, Ab4 N752 or Ab4 gD4. Each data point (open circle) represents a tissue sample of an individual horse. An asterisk indicates significant lower affinity compared among groups (*p* < 0.001). The affinity is defined by the number of positive samples in combination with viral loads. Bars represent the mean. Note: data points at the zero-line (x-axis) represent samples where EHV-1 DNA was not detected despite neuronal cells or lymphocytes having been observed microscopically.

As some samples were lost with shipping or did not contain nerve cell bodies upon microscopic screening, tissue samples available included: 22 TG, seven DRG, and 70 S/PS ganglia from head, neck, thorax or abdomen (see Figure 1). TG and DRG were positive for viral gDNA in 45% (10/23) and 100% (7/7) of total samples, respectively. TG samples had mostly low copy numbers of viral gDNA, while six of seven samples of DRG had high copy numbers of viral gDNA. The only parasympathetic ganglion collected, the ciliary ganglion, had the lowest count of positive samples with three out of 12 samples. Sympathetic ganglia from the head and neck, abdomen and ST showed similar results, and about half of the samples in each group tested positive for viral gDNA. Interestingly, one of the positive samples in the Ab4 gD4 group had very high copy numbers, resulting in a higher mean of viral loads compared to the other groups. However, the affinity (number of positive samples in combination with viral loads) in that group was significantly lower. Worth mentioning, ST had most of the ‘high positive’ samples of all S/PS (all Figure 1 and Figure 4).

### 2.3. cDNA and IHC Results

All samples regardless of their viral gDNA status were investigated for transcriptional activity of the L gene. L mRNA was detectable in none of the samples. Additionally, all viral gDNA positive samples were labeled using specific IHC. No viral protein was detectable.

## 3. Discussion

In the present study, EHV-1 gDNA was detected in a variety of neural and lymphatic tissues throughout the body at 70 dpi. This extends our current knowledge about EHV-1 and shows additional sites of latency beyond the TG and RALT including the mesenteric lymph nodes and spleen, as well as a variety of somatic sensory and S/PS ganglia. Late gene expression and specific EHV immunolabeling were consistently absent further suggesting the viral DNA to be a result of an arrested, potentially latent stage of EHV-1 chronic-persistent infection.

The TG as well as the mandibular and retropharyngeal lymph nodes have long been reported sites of EHV-1 latency [1,3,4,6]. Our findings are consistent with these reports. Furthermore, detection of EHV-1 Ab4 WT in the TG and the retropharyngeal lymph node has been recently described [6]. Previously, the bronchial lymph node of horses has also been described as a site of EHV-1 latency [4]. Interestingly, our study shows that while we detected EHV-1 gDNA in this location, the bronchial lymph node was the least likely location of the RALT group to contain EHV-1 gDNA. On the other hand, our study reports EHV-1 gDNA detection in the equine pharyngeal roof, which has not been previously shown. These latter findings are consistent with those shown for other members of the Alphaherpesvirinae, such as BoHV-1 and SHV-1, which have been detected in pharyngeal lymphoid tissue of latently infected calves and piglets respectively, suggesting that the tonsil is a site for viral persistence [10,11,12,13]. It is plausible that the pharyngeal roof with many primary lymph follicles and the secondary lymph nodes of the upper respiratory tract are major sites for EHV-1 chronic-persistent infection in horses, however, follow-up studies in a larger number of horses of different ages, breeds and at different stages following primary infection or reactivation should examine this question further. Of interest, with this site would be the proximity to the upper respiratory epithelium, as it would allow for quick reactivation and viral nasal shedding. However, the route of infection, dose of viral inoculum and nasopharyngeal instillation of the inoculum, could also be the reason that the lymphoid tissue of the pharyngeal roof and the draining lymph nodes of the upper respiratory tract are primary sites of viral DNA recovery in our study, rather than the further physically distanced bronchial lymph node.

The detection of EHV-1 gDNA in the spleen and mesenteric lymph node samples collected 70 dpi is a novel finding. However, the presence of the EHV-1 Ab4 WT strain in the mesenteric lymph node 70 dpi has been described previously by Giessler et al. from our group [6]. Interestingly, the spleen has been described as a site of BoHV-1 latency in experimentally infected cattle [17]. Until now, BoHV-1 was the only Alphaherpesvirus member in which splenic persistence had been documented and it has been shown that in this case immunocompetent lymphocytes reach the spleen via blood vessels and proliferate locally [18]. During viremia, EHV-1 gDNA has been identified in CD8+ T-lymphocytes, B-lymphocytes, monocytes, and less frequently in CD4+ T-lymphocytes of experimentally infected horses [19]. This finding suggests that during a productive EHV-1 viremia, the virus may reach the spleen and could be retained in splenic germinal centers during later stages of infection. Alternatively, the detection of EHV-1 gDNA in splenic tissue may be due to its presence in circulating persistently infected lymphocytes rather than true localization to splenic tissue. Furthermore, lymphocytes home repeatedly to secondary lymphoid organs, including the spleen and lymph nodes, reside in these organs transiently, and return to the circulation [18]. In addition, in this study, all but one horse had EHV-1 gDNA positive mesenteric lymph nodes that were also positive in various other RALT and/or spleen. Although viral gDNA was present in a variety of lymphoid tissue, PBMC isolated on Day 70 were negative for viral gDNA in all but one horse. This was in contrast to several other studies that have reported persistent EHV-1 infection in PBMC and might be explained by the fact that EHV-1 DNA is only present at very low levels, which were not detected with our assay system. Alternatively, presence in circulating PBMC maybe more intermittent or depend on how long ago a previous infection or reactivation has occurred. It remains to be determined whether the abdominal lymphoid tissues represent a true localization for EHV-1 during viral persistence or if this finding is a consequence of recirculation of infected lymphocytes and therefore a transient phenomenon.

In addition to the identification of various lymphoid tissues, this study also identified novel sites for EHV-1 persistence or latency in a variety of neural tissues. Localization of latency in the TG is well-documented for EHV-1 and assumed to occur following retrograde axonal transport along the trigeminal nerve after primary infection of the nasopharyngeal mucosa. However, the mechanisms through which EHV-1 reached various other neural locations throughout the body in the subjects of this study remains unknown. The presence of EHV-1 gDNA in the ciliary (GCi) and cranial cervical (GCcr) ganglia could be due to the close proximity of primary viral replication in the conjunctiva or in the guttural pouch (GP) epithelium. The virus may establish persistence at these sites using retrograde axonal transport, similar to the TG pathway. These findings are consistent with a recent publication where FeHV-1 was detected in a variety of neural tissues of experimentally infected cats [8,9]. Alternatively, in particular, the detection of primary lymphoid follicles of the GP epithelium and the proximity of enrolled GCcr preparations in this study could have been the cause for false-positive results in the GCcr tissue analysis.

However, EHV-1 gDNA was also detected in the farther distanced caudal cervical (GCca), mesenteric (GM) and celiac (GCoe) ganglia, as well as in the ST and DRG. Please note, that EHV-1 Ab4 WT in the ST 70 dpi has been demonstrated previously [6]. As these sites are not in the vicinity to the primary site of infection, it is not clear how the virus reached these ganglia to establish a putative latent infection. The presence of VZV, a close relative to EHV-1, has also been demonstrated in various sensory and autonomic ganglia in addition to TG [7,20,21,22,23,24,25,26]. Furthermore, there is growing evidence that VZV most likely uses infected lymphocytes as vehicles to reach ganglion cells during cell-associated viremia [27,28,29]. A study by Giessler et al., 2020 from our group recently showed EHV-1 gDNA positive in-situ hybridization (ISH) signal in support or interstitial cells that gather around neural cell bodies in ganglia by Day 30 p.i. and CD3+ T-lymphocytes could be located frequently in close proximity to the neuronal cell body using IHC. In contrast, ISH positive neural cell bodies were seen by Day 70 p.i. [6]. Although the non-neural ISH signal could not be definitely attributed to either lymphocytes or peripheral glial cells (satellite glial cells), these findings combined with the numerous EHV-1 gDNA positive abdominal neural tissues detected in the present study, suggest viremia as an alternative route for latency establishment for EHV-1.

Viremia is also crucial to EHV-1 pathogenesis and the prerequisite for complications of abortion or myelopathy. Our findings are highly suggestive that this route also plays a role in the transport of virus to distant neural sites, rather than axonal transportation. Viral DNA has been detected in all PBMC subpopulations of experimentally infected horses during cell-associated viremia of acute infection followed by lymphocytic/monocytic extravasation or endothelial (contact-induced) infection [19,30]. An alternative explanation could be that our detected viral DNA remains in the endothelial cells of arterioles, venules, or support cells in the vicinity to ganglia or within perivascular mononuclear cells, as previously described during lytic infection of the uterine tissue and the spinal cord [31,32]. At this point we have yet to elucidate whether EHV-1 gDNA is present in ganglion cells; in ganglion support (or satellite) cells, or in infiltrative leukocytes or histiocytes. Our attempts to correlate absent/low, moderate or high lymphocytic/monocytic infiltration with results of EHV-1 gDNA quantification appeared not sensitive enough to provide an answer to this question, as most of the neural parenchyma contained only mild lymphocytic infiltrates.

EHV-1 latency defined as gDNA and latency associated transcripts (LAT) as mRNA have been reported to be established by Day 21 p.i. [1]. Samples in the present study were obtained around Day 70 p.i. As expected, this was enough time to establish a chronic-persistent, non-lytic/arrested, potentially latent EHV-1 infection in the animals of this study. EHV-1 latency has previously been defined as a stage characterized by absence of viral replication, where transcriptional activity is limited to a region antisense to the immediate early (IE) gene [1,2,5,33,34,35]. In this study, we assumed EHV-1’s arrested/latent state when a sample was EHV-1 gDNA positive while simultaneously, late (L) gene mRNA expression and (lytic) protein expression was absent [4,36,37].

Efstathiou et al. (2005) described the several weeks p.i. phase of HSV-1 infection as an ongoing process of transition from simultaneous low-grade lytic and latent infection activity in different cells of the same tissue. Their study suggests that cells with lytic replication will go into apoptosis, while those with harboring latent virus will survive. However, the latter cells are in a hostage situation where latent virus can reactivate and cause de novo infection and initiate renewed spread. Our results suggest that EHV-1 is present at multiple sites throughout the body, in an arrested and very restricted, quiet phase of infection. However, it is still possible that EHV-1 is still replicating at a very low level at 70 dpi, and that the replicative pace may differ between sites. The simultaneous presence of a lymphocytic/monocytic infiltrate in basically most of the neural samples in our study could be an indicator of an actively involved immune system to clear infection at these sites and shows the limitation in our observations to a single p.i. time point. However, the wide-spread distribution pattern in the horses in this study highlights the complexity of EHV-1’s chronic-persistent infection and suggests that a onetime limited tissue analysis close to primary sites of replication is probably insufficient for determination of a true latency status diagnosis in an individual horse.

Comparison between groups was used to determine the presence of tissue preferences among EHV-1 Ab4 WT and the two mutants with assumed differing neuropathogenic potential and significant differences in viremia. Interestingly, we showed a clear difference in the distribution of EHV-1 gDNA between the groups. The polymerase mutant (Ab4 N752) caused clinically significantly different disease compared to Ab4 WT and Ab4 gD4. Infection with this EHV-1 Ab4 N752 mutant caused the most severe respiratory clinical signs after infection [16]. However, viral DNA was found in less than 10% of the analyzed RALT samples in this group. A study by Quintana et al. (2011), where the equine airway epithelium was assessed immunologically in vitro, suggested that early events in the respiratory tract may shape downstream responses and clinical outcome [38,39]. In addition, local virus-specific mucosal immunity is believed to represent a first line of defense against EHV-1 infection and may impede viremia [40]. In the present study, horses infected with Ab4 N752 did not show a significant secondary fever and exhibited significantly less viremia. Nevertheless, EHV-1 gDNA was also detected in various neural tissues, suggesting that chronic-persistent infection reached these sites rather effectively. The exact mechanisms by which different sites of latency were reached, and potential cell types for which EHV-1 gDNA spread to ganglionic samples requires further investigation.

Horses infected with EHV-1 Ab4 WT showed significantly higher viremia than the other groups. Three horses in this group developed clinical signs of EHM. None of the horses in the other groups displayed signs of EHM [16]. Despite the absence of severe clinical signs in horses infected with EHV-1 Ab4 gD4, and the possible role of gD4 in disease attenuation, no significant differences in viral genome distribution were identified between EHV-1 Ab4 gD4 and EHV-1 Ab4 WT. In both groups, viral gDNA could be detected in neural and lymphoid tissues throughout the horses’ body. Overall, it is likely that EHV-1 uses both hematogenous and neural (axonal) spread for establishment of persistence/latency but that there may be differences between viral strains and mutants, and between EHV-1 and EHV-4 in whether hematogenous or neural spread is the preferred route.

Some of the limitations of the study are the lack of a control group, which was not included due to ethical reasons. In addition, parallel infection of all three groups would have been ideal to provide the exact same experimental conditions, but implementation was not possible due to human resources and available facilities. Sample collection of the correct part of the DRG nerve root in a time sensitive containment of necropsy proved to be difficult due to rather disperse nature of DRG in equids. To increase neuroanatomical accuracy in future, sampling protocols have been amended. Most importantly, our results represent a single time point (70 dpi), which, ideally should be compared to one or more even later time points p.i. (months/year), although this is highly impracticable.

## 4. Materials and Methods

### 4.1. Animals

A total of 25 Western Stock yearling horses (14 colts and 11 fillies, age: 10–15 months) were infected on three separate occasions as described elsewhere between June 2014 and Mai 2015 [16,41]. The study was approved by the institutional animal care and use committee of Michigan State University, MI, USA, and details of this study are published by Holz et al. 2017 and 2019 [16,41].

### 4.2. Experimental Design

A total of 25 Western Stock yearling horses were divided into three groups with 8, 9 and 8 subjects per group. All horses had serum neutralizing antibody titers against EHV-1 and EHV-4 of less than 1:4 and 1:40, respectively. Horses were infected via pharyngeal instillation of 5 × 10^7^ TCID50/mL with either wild type EHV-1, neuropathogenic strain Ab4 suspension (group Ab4 WT; n = 8), an Ab4 mutant with aspartate (D) substituted by asparagine (N) at position 752 (Ab4 N752; n = 9), or an Ab4 mutant where the EHV-1 glycoprotein D (gD) nucleotide sequence was replaced by EHV-4 gD equivalent (Ab4 gD4; n = 8) [9,12,13].

At Day 70 (±3 days) p.i. venous blood was collected in heparinized tubes following sedation with detomidine (0.012 mg/kg i.v.) and prior to euthanasia with an intravenous pentobarbital overdose (380 mg/kg i.v.). PBMC were isolated using ficoll hypaque gradient centrifugation and were stored in liquid nitrogen until further analysis. A postmortem examination and sample collection was carried out immediately and only two or three horses were euthanized per day to allow for a comprehensive postmortem examination and tissue sampling. All horses of a particular group (Ab4 WT; Ab4 N752; or Ab4 gD4) were processed within one week.

### 4.3. Tissue Collection

Tissue collected included mandibular and retropharyngeal lymph nodes, pharyngeal roof (Waldeyer’s ring) and bronchial lymph node, as representatives of RALT, as well as mesenteric lymph node and spleen. In addition to the TG, the spinal DRG of the lumbar spinal cord, as well as S/PS ganglia from the head, neck, thorax and abdomen (ciliary, cranial and caudal cervical ganglion, mesenteric/celiac ganglion and sympathetic trunk) were collected. All tissues were fixated in 10% neutral-buffered formalin and embedded in paraffin after 24–36 h of fixation and subsequent trimming.

### 4.4. Tissue Processing

Tissue blocks were sectioned at 4 µm thickness. Prior to sectioning the microtome was treated with RNase killer solution spray (RNase ZAP™, Sigma-Aldrich, Darmstadt, Germany). Six serial sections were cut, with the third section set aside for H&E staining. The remaining five sections were placed in a sterile, RNAse free Eppendorf tube and stored at −80 °C until further processing.

H&E stained neural tissue slides were evaluated for presence of ganglion (neuronal) cell bodies using light microscopy. Where no ganglion cells were identified, paraffin blocks were sectioned further, (six cuts with Cut #3 for light microscopy assessment) until ganglion cells were identified, or for up to a total of three attempts. Sections that did not contain ganglion cells were excluded from further analyses and are marked ‘no ganglion cells’ in Figure 1. Where ganglion cells were present, the sections were scored by a veterinary neuropathologist (K.M.) for histopathological changes, cell degeneration and mononuclear cell infiltration, using a scoring key of no changes, mild, moderate, or severe changes (Table S1).

### 4.5. Quantitative PCR (qPCR)—Genomic and Reverse Transcriptase PCR (RT-PCR)

For determination of ‘latent’ EHV-1, collected tissues were screened for both genomic viral DNA (gDNA) and viral mRNA of a transcriptional product of the viral late (L) gene as previously described [33]. This method associates the identification of viral gDNA in the absence of transcriptional activity (absence of viral mRNA), with the confirmation of EHV-1 in its non-replicative ‘latent’ phase (chronic-persistent infection), while detection of both gDNA and L-gene mRNA is interpreted as ‘lytic’ EHV-1 being present (replication phase) [4,36].

Total DNA and RNA were extracted from formalin- fixed, paraffin-embedded (FFPE) sample sections using AllPrep DNA/RNA FFPE Kit (Qiagen, Hilden, Germany) following the manufacturer′s instructions. Total DNA and RNA were extracted from PBMC samples using the QIAamp DNA Blood Mini Kit and RNeasy Mini Kit, respectively, following the manufacturer′s instructions, (Qiagen, Hilden, Germany). DNA/RNA free water was included as extraction control in each extraction.

RNA extracted samples were tested to confirm absence of gDNA by real-time qPCR using the housekeeping gene equine glyceraldehyde-3-phosphate dehydrogenase (eqGAPDH) as a marker [42]. Samples that were eqGAPDH gDNA positive were treated with the RQ1 RNase-free DNase Kit (Promega) following the manufacturer’s instructions, to remove any remaining gDNA. Once the absence of gDNA was confirmed, the RNA concentration was measured by nanodrop (NanoDrop™ Spectrophotometer ND-1000, Peqlab Biotechnologie GmbH, Erlangen, Germany). Subsequently, up to 5 µg of RNA was reverse transcribed using the QuantiNova Reverse Transcription Kit (Qiagen, Hilden, Germany) with random primers, following the manufacturer’s instructions. To assess if the reverse transcription was successful, the QuantiNova Internal Control RNA (QN IC RNA) was included in each reaction according to manufacturer′s recommendations. The cDNA was further diluted 1:5 with RNase/DNase-free water and cDNA samples were stored at −20 °C until further analysis.

Unless stated otherwise, all qPCR reactions for DNA and cDNA analysis were performed with the same thermal profile including an initial 95 °C step for 2 min, followed by 40 cycles of 95 °C for 10 s and 60 °C for 60 s (and hold 60 °C for 60 s). The qPCR was performed in a total reaction volume of 20 µL using 1 x SensiFAST™ Probe Lo-ROX Kit (Bioline, Luckenwalde, Germany) and 5 µL of the template. Amplification and detection were performed in strips of eight PCR tubes and caps (BRAND^®^, Wertheim, Germany) using the Stratagene Mx3000P cycler (Agilent Technologies, Waldbronn, Germany). All reactions included a non-template control (DNA/RNA-free water), an extraction control and a positive control for EHV-1 (EHV-1 gDNA extracted from EHV-1 infected RK13 cells), and an extraction control for equine tissue (DNA extracted from equine liver).

For detection of EHV-1 gDNA and of reverse transcribed L gene cDNA in tissue samples, a qPCR targeting a region of the glycoprotein B gene within the open reading frame 33 (ORF 33) was performed as previously published [43]. Forward and reverse primers were added to a final concentration of 450 nM each, and the probe to a final concentration of 100 nM. For absolute quantitation and generation of a standard curve a tenfold dilution series of a defined EHV-1 DNA plasmid (courtesy of W. Azab and N. Osterrieder) was tested [15]. The number of cells was estimated using beta-2-microglobulin (B2M) as a housekeeping gene extrapolated to a standard curve generated with oligonucleotides specific to equine B2M [34]. Viral DNA and mRNA concentrations were expressed as copies per million cells, considering that each diploid eukaryotic cell contains two copies of the B2M gene [33,44]. All primers and probes sequences are presented in Table 1.

### 4.6. EHV-1 Specific Immunohistochemistry

As a further control for translational gene activity, which would be expected in lytic infection activity, all gDNA-positive/cDNA-negative tissue sections were further assessed immunohistochemically. An EHV-1/ERV polyclonal caprine antiserum (VMRD, Pullman, WA, USA) was used to detect (presumably lytic) virus protein expression. EHV-1 gDNA PCR positive lung tissue from an EHV-1 aborted fetus was used as positive control, and PCR negative lung from archived samples was used as negative control. Sections were processed according to an established EHV-1 IHC protocol. In short: following deparaffinization, tissue sections were mounted on slides and antigen retrieval was performed with citrate buffer (0.1 M, pH 6.0) and heating in a microwave oven (700 W, 20 min). Samples were incubated with rabbit antiserum (1:10, 30 min), directly followed by incubation with the polyclonal caprine EHV-1/ERV antiserum (1:1600, 60 min) without washing. After incubation, slides were rinsed with tris buffered saline (TBS) and incubated with a secondary rabbit anti-goat biotinylated antibody (Vector Laboratories LTD, Burlingame, CA, USA) conjugated to horseradish peroxidase, incubated with avidin-biotin-complex (1:100, 30 min) and visualized by 3,3′- diaminobenzidine (DAB) (Vector laboratories LTD, Burlingame, CA, USA). Samples were rinsed with running tap water to stop detection and counterstaining was performed with filtered Mayer’s haemalaun (AppliChem GmbH, Darmstadt, Germany).

### 4.7. Statistical Analysis

Statistical analysis was performed using IBM-SPSS-Statistics 24.0 software (IBM Deutschland GmbH, Ehningen, Germany). Differences between viral mutant distribution pattern in tissues (the affinity to neural and/or lymphoid tissues) within each group and among groups were compared using cross tabulation and U tests for group ranked data. The affinity of the virus to a certain tissue type was defined by the frequency and the viral genomic load found in that tissue type. The correlation coefficient of tissue qPCR results and degree of lymphocytic/histiocytic influx was calculated using cross tabulation and chi-squared test. The Mann–Whitney U test was used to analyze the correlation between cell number and qPCR results. *p*-values < 0.05 were considered statistically significant.

## 5. Conclusions

EHV-1 gDNA without detectable L gene transcriptional and translational activity was confirmed in various neural and lymphoid tissues of experimentally infected horses. These findings provide strong evidence of a non-lytic, persistent, likely latent infection in horses euthanized 70 dpi. Results further suggest that viral chronic persistence occurs in additional tissues than those reported previously. A random distribution pattern and differences in tissue tropism among viral mutants of differing neuropathogenic potential and propensity to establish viremia highlights the complexity of EHV-1 infection and the need for further investigations.

Cell-associated viremia besides retrograde axonal transportation may play a pivotal role in distribution. In addition, it is important to keep in mind that our findings are a ‘moment-in-time’ observation with possible redistribution or clearance of virus with time. Future research is needed to ascertain if tissue tropism of different EHV-1 isolates differs, and to determine if the additional locations, identified in this study, are true and long-term sites of EHV-1 chronic-persistent infection.

## Figures and Tables

**Table 1:** Primers and probes used for analyses.

eGAPDH	
eGAPDH (F)	5′-GCCATCACTGCCACCCAG-3′
eGAPDH (R)	5′-TGGCAGCACCAGTAGAAGCA-3′
eGAPDH (probe)	5′[6FAM]-AGGGGCTGCCCAGAACATCATCC–[TAMRA]3′
B2M	
B2M (F)	5′-ATGGAAAGCCAAATTTCCTG-3′
B2M (R)	5′-ACCGGTCGACTTTCATCTTC-3′
B2M (probe)	5′[HEX]-TGGGTTCCATCCGCCTGAGA–[BHQ1]3′
gB (L gene)	
gB (F)	5′- CATACGTCCCTGTCCGACAGAT-3′
gB (R)	5′- GGTACTCGGCCTTTGACGAA-3′
gB (probe)	5′[FAM]- GGTACTCGGCCTTTGACGAA–[BHQ1]3′

## Data Availability

All datasets generated for this study are included in the article/supplementary materials.

## Supplementary Materials

The following are available online at https://www.mdpi.com/article/10.3390/pathogens10060707/s1, Table S1: scoring key for histopathological slide evaluation: cell-pathology and cellular infiltration.

## References

[1] Slater J., Long M. (2014). Equine Herpesviruses. Equine Infectious Diseases.

[2] Efstathiou S., Preston C.M. (2005). Towards an understanding of the molecular basis of herpes simplex virus latency. Virus Res..

[3] Slater J.D., Borchers K., Thackray A.M., Field H.J. (1994). The trigeminal ganglion is a location for equine herpesvirus 1 latency and reactivation in the horse. J. Gen. Virol..

[4] Pusterla N., Mapes S., David Wilson W. (2012). Prevalence of latent alpha-herpesviruses in Thoroughbred racing horses. Vet. J..

[5] Chesters P.M., Allsop R., Purewal A., Edington N. (1997). Detection of latency-associated transcripts of equid herpesvirus 1 in equine leukocytes but not in trigeminal ganglia. J. Virol..

[6] Giessler K.S., Samoilowa S., Soboll Hussey G., Kiupel M., Matiasek K., Sledge D.G., Liesche F., Schlegel J., Fux R., Goehring L.S. (2020). Viral Load and Cell Tropism During Early Latent Equid Herpesvirus 1 Infection Differ Over Time in Lymphoid and Neural Tissue Samples From Experimentally Infected Horses. Front. Vet. Sci..

[7] Gilden D.H., Gesser R., Smith J., Wellish M., Laguardia J.J., Cohrs R.J., Mahalingam R. (2001). Presence of VZV and HSV-1 DNA in Human Nodose and Celiac Ganglia. Virus Genes.

[8] Parzefall B., Schmahl W., Fischer A., Blutke A., Truyen U., Matiasek K. (2010). Evidence of feline herpesvirus-1 DNA in the vestibular ganglion of domestic cats. Vet. J..

[9] Townsend W.M., Jacobi S., Tai S.-H., Kiupel M., Wise A.G., Maes R.K. (2013). Ocular and neural distribution of feline herpesvirus-1 during active and latent experimental infection in cats. BMC Vet. Res..

[10] Winkler M.T.C., Doster A., Jones C. (2000). Persistence and Reactivation of Bovine Herpesvirus 1 in the Tonsils of Latently Infected Calves. J. Virol..

[11] Brockmeier S.L., Lager K.M., Mengeling W.L. (1993). Comparison of in vivo reactivation, in vitro reactivation, and polymerase chain reaction for detection of latent pseudorabies virus infection in swine. J. Vet. Diagn. Investig..

[12] Perez S., Inman M., Doster A., Jones C. (2005). Latency-related gene encoded by bovine herpesvirus 1 promotes virus growth and reactivation from latency in tonsils of infected calves. J. Clin. Microbiol..

[13] Romero C.H., Meade P.N., Homer B.L., Shultz J.E., Lollis G. (2003). Potential sites of virus latency associated with indigenous pseudorabies viruses in feral swine. J. Wildl. Dis..

[14] Nugent J., Birch-Machin I., Smith K., Mumford J., Swann Z., Newton J., Bowden R., Allen G., Davis-Poynter N. (2006). Analysis of equid herpesvirus 1 strain variation reveals a point mutation of the DNA polymerase strongly associated with neuropathogenic versus nonneuropathogenic disease outbreaks. J. Virol..

[15] Azab W., Osterrieder N. (2012). Glycoproteins D of equine herpesvirus type 1 (EHV-1) and EHV-4 determine cellular tropism independently of integrins. J. Virol..

[16] Holz C.L., Nelli R.K., Wilson M.E., Zarski L.M., Azab W., Baumgardner R., Osterrieder N., Pease A., Zhang L., Hession S. (2017). Viral genes and cellular markers associated with neurological complications during herpesvirus infections. J. Gen. Virol..

[17] Mweene A., Okazaki K., Kida H. (1996). Detection of Viral Genome in Non-Neural Tissues of Cattle Experimentally Infected with Bovine Herpesvirus 1. Jpn. J. Vet. Res..

[18] Butcher E.C., Picker L.J. (1996). Lymphocyte homing and homeostasis. Science.

[19] Wilsterman S., Soboll-Hussey G., Lunn D., Ashton L., Callan R., Hussey S., Rao S., Goehring L. (2011). Equine herpesvirus-1 infected peripheral blood mononuclear cell subpopulations during viremia. Vet. Microbiol..

[20] Gilden D.H., Vafai A., Shtram Y., Becker Y., Devlin M., Wellish M. (1983). Varicella-zoster virus DNA in human sensory ganglia. Nature.

[21] Gilden D.H., Rozenman Y., Murray R., Devlin M., Vafai A. (1987). Detection of varicella-zoster virus nucleic acid in neurons of normal human thoracic ganglia. Ann. Neurol..

[22] Furuta Y., Takasu T., Fukuda S., Sato-Matsumura K.C., Inuyama Y., Hondo R., Nagashima K. (1992). Detection of varicella-zoster virus DNA in human geniculate ganglia by polymerase chain reaction. J. Infect. Dis..

[23] Mahalingam R., Wellish M.C., Dueland A.N., Cohrs R.J., Gilden D.H. (1992). Localization of herpes simplex virus and varicella zoster virus DNA in human ganglia. Ann. Neurol..

[24] Kennedy P.G.E., Grinfeld E., Gow J.W. (1998). Latent varicella–zoster virus is located predominantly in neurons in human trigeminal ganglia. Proc. Natl. Acad. Sci. USA.

[25] Kennedy P.G.E., Grinfeld E., Gow J.W. (1999). Latent Varicella-Zoster Virus in Human Dorsal Root Ganglia. Virology.

[26] Hyman R., Ecker J., Tenser R. (1983). Varicella-zoster virus RNA in human trigeminal ganglia. Lancet.

[27] Grigoryan S., Yee M.B., Glick Y., Gerber D., Kepten E., Garini Y., Yang I.H., Kinchington P.R., Goldstein R.S. (2015). Direct transfer of viral and cellular proteins from varicella-zoster virus-infected non-neuronal cells to human axons. PLoS ONE.

[28] Zerboni L., Arvin A.M. (2016). The Pathogenesis of Varicella-Zoster Virus Neurotropism and Infection. Neurotropic Viral Infections.

[29] Depledge D.P., Sadaoka T., Ouwendijk W.J.D. (2018). Molecular Aspects of Varicella-Zoster Virus Latency. Viruses.

[30] Goehring L., Hussey G., Ashton L., Schenkel A., Lunn D. (2011). Infection of central nervous system endothelial cells by cell-associated EHV-1. Vet. Microbiol..

[31] Smith K., Borchers K. (2001). A study of the pathogenesis of equid herpesvirus-1 (EHV-1) abortion by DNA in-situ hybridization. J. Comp. Pathol..

[32] Stierstorfer B., Eichhorn W., Schmahl W., Brandmüller C., Kaaden O.R., Neubauer A. (2002). Equine herpesvirus type 1 (EHV-1) myeloencephalopathy: A case report. J. Vet. Med. Ser. B.

[33] Pusterla N., Wilson W.D., Mapes S., Finno C., Isbell D., Arthur R.M., Ferraro G.L. (2009). Characterization of viral loads, strain and state of equine herpesvirus-1 using real-time PCR in horses following natural exposure at a racetrack in California. Vet. J..

[34] Abdelgawad A., Damiani A., Ho S.Y.W., Strauss G., Szentiks C.A., East M.L., Osterrieder N., Greenwood A.D. (2016). Zebra Alphaherpesviruses (EHV-1 and EHV-9): Genetic Diversity, Latency and Co-Infections. Viruses.

[35] Baxi M.K., Efstathiou S., Lawrence G., Whalley J.M., Slater J.D., Field H.J. (1995). The detection of latency-associated transcripts of equine herpesvirus 1 in ganglionic neurons. J. Gen. Virol..

[36] Pusterla N., Mapes S., Wilson W.D. (2010). Prevalence of equine herpesvirus type 1 in trigeminal ganglia and submandibular lymph nodes of equids examined postmortem. Vet. Rec..

[37] Slater J. (2017). Personal Communication.

[38] Quintana A.M., Landolt G.A., Annis K.M., Hussey G.S. (2011). Immunological characterization of the equine airway epithelium and of a primary equine airway epithelial cell culture model. Vet. Immunol. Immunopathol..

[39] Hussey G.S., Ashton L.V., Quintana A.M., Van de Walle G.R., Osterrieder N., Lunn D.P. (2014). Equine herpesvirus type 1 pUL56 modulates innate responses of airway epithelial cells. Virology.

[40] Breathnach C., Yeargan M., Timoney J., Allen G. (2006). Detection of equine herpesvirus-specific effector and memory cytotoxic immunity in the equine upper respiratory tract. Vet. Immunol. Immunopathol..

[41] Holz C.L., Sledge D.G., Kiupel M., Nelli R.K., Goehring L.S., Soboll Hussey G. (2019). Histopathologic Findings Following Experimental Equine Herpesvirus 1 Infection of Horses. Front. Vet. Sci..

[42] Pusterla N., Wilson W.D., Conrad P.A., Barr B.C., Ferraro G.L., Daft B.M., Leutenegger C.M. (2006). Cytokine gene signatures in neural tissue of horses with equine protozoal myeloencephalitis or equine herpes type 1 myeloencephalopathy. Vet. Rec..

[43] Hussey S.B., Clark R., Lunn K.F., Breathnach C., Soboll G., Whalley J.M., Lunn D.P. (2006). Detection and Quantification of Equine Herpesvirus-1 Viremia and Nasal Shedding by Real-Time Polymerase Chain Reaction. J. Vet. Diagn. Investig. Off. Publ. Am. Assoc. Vet. Lab. Diagn. Inc..

[44] Allen G., Kydd J., Slater J., Smith K. (2004). Equid Herpesvirus-1 (EHV-1) and-4 (EHV-4) Infections. Infectious Diseases of Livestock.

