# Supplementary material for: Equid herpesvirus-1 Distribution in Equine Lymphoid and Neural Tissues 70 Days Post Infection
Source: Pathogens. 2021 Jun 5;10(6):707. doi: 10.3390/pathogens10060707 (PMC8228440; doi:10.3390/pathogens10060707)
Supplement: Supplementary file 1 [file pathogens-10-00707-s001.zip › pathogens-1215383-supplementary.pdf]

Table S1: Scoring key for histopathological slide evaluation: cell-pathology and cellular infiltration

	**ganglion cells**			
**ganglion cells**	density	normal= 0	reduced= 1	increased= 1
	size	small= 1	normal= 0	large= 1
	neuronal cluster	none= 0	sporadic= 1	predominant= 2
	**cytoplasm**			
	Nissl substance	not homogenised = 0	coarse segregation= 1	chromatolysis =2
	cytoplasmic inclusions	absent= 0	present= 3<br>if present, type of inclusion:	
	**nucleus with nucleolus**			
	position	central= 0	paracentral= 1	
	vacuolization	absent= 0	present= 2	
	intranuclear inclusions	absent= 0	present= 3<br>if present, type of inclusion:	
	**satellite cells**			
**satellite cells**	cell layers	single-row= 0	multiple= 2	
	hypertrophy & hyperplasia	absent= 0	present= 3	
	Nageotte´s bodies	absent= 0	occasional=1 (≤3)	multiple=2 (>3)
	**lymphocytic infiltration**			
**inflammation**	position	perivascular	perineuronal	diffuse interstitial
	intensity	mild= 1	moderate= 2	severe= 3
	distribution	occasional= 1	multifocal= 2	diffuse= 3
	neuronophagia	absent= 0	present= 2	
	**nerve fibres**			
**nerve fibres**	Myelin changes	absent= 0	present= 2<br>if present, type of changes:	
	Wallerian like degeneration	absent= 0	individual=1	multiple=2
	axonal spheroids	absent= 0	individual=1	multiple=2

Scoring key for histopathological slide evaluation: cell-pathology and cellular infiltration of neural tissue sections where ganglion cells were present. For each group of characteristics (ganglion cells, satellite cells, inflammation and nerve fibres) the points of the most severe change were crucial for the total points. A maximum score of 12 points was possible, with 0 points indicating no changes, 1-4 points mild, 5-8 points moderate and 9-12 points severe changes.
